# Supplementary material for: Environmentally enriched pigs have transcriptional profiles consistent with neuroprotective effects and reduced microglial activity
Source: Behav Brain Res. 2018 Sep 17;350:6–15. doi: 10.1016/j.bbr.2018.05.015 (PMC6002610; doi:10.1016/j.bbr.2018.05.015)
Supplement: Supplementary file 1 [file mmc1.docx]

1. **Supplemental Information**

**Figure S1**: Cluster graphs from 1 hour Cluster lists found in Table S3 that show clear segregation between EE and B animals. Panel (A) Microglia and connective tissue genes; Panel (B) Immune and connective tissue genes; Panel (C) IEG; Panel (D) All clusters. Each column represents a single animal with B house pigs as columns 1-6 and EE pigs columns 7-12. There is clear segregation in panel D of the EE animals (top clusters) and B animals (bottom clusters)

**Table S1**: Means and SEM of behaviours (as percentage of time observed) at all time-points by treatment.

**Table S2**: Differentially expressed genes at 1 and 4 hours. With log2 fold change, p value, average counts and average transcripts per million for each treatment group.

**Table S3**: Genes present in gene-gene cluster analysis.

**Table S4**: IEG and microglial reference gene list. Reference list used for randomisation tests.
